# Supplementary material for: Integrative Analysis of Transcriptomic and Lipidomic Profiles Reveals a Differential Subcutaneous Adipose Tissue Mechanism among Ningxiang Pig and Berkshires, and Their Offspring
Source: Animals (Basel). 2023 Oct 25;13(21):3321. doi: 10.3390/ani13213321 (PMC10647668; doi:10.3390/ani13213321)
Supplement: Supplementary file 1 [file animals-13-03321-s001.zip › animals-2592124-supplementary.pdf]

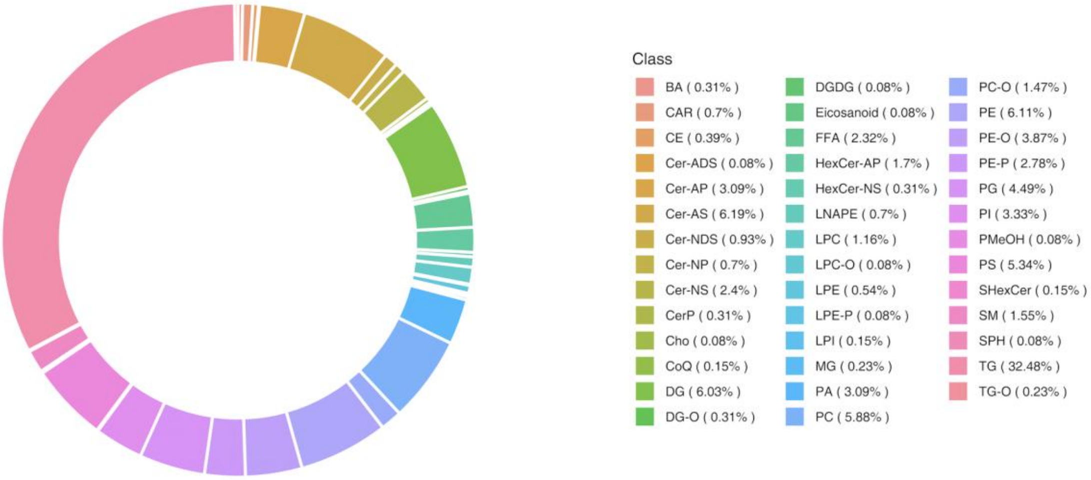

Figure S1. Ring diagram of lipid subclass composition, 18 samples of subcutaneous adipose from six Ningxiang pigs, six Berkshires and six F1 pigs, respectively, were used to detect lipidomic profile.

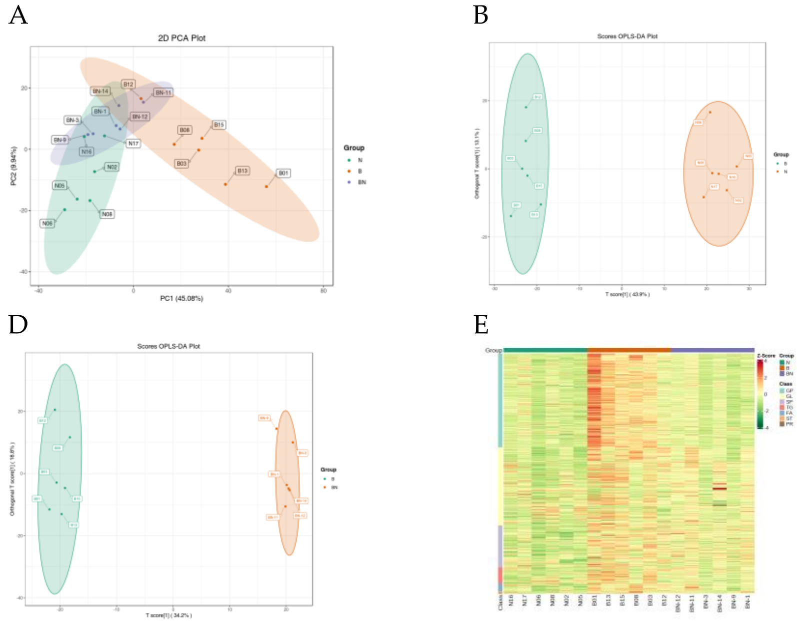

Figure S2. The subcutaneous adipose tissue lipid composition. (A) PCA of the subcutaneous adipose tissue from Ningxiang pigs, Berkshires and F1 offspring. (B) OPLS-DA of the subcutaneous adipose tissue from Ningxiang pigs and Berkshires. (C) OPLS-DA of the subcutaneous adipose tissue from Ningxiang pigs and F1 offspring. (D) OPLS-DA of the subcutaneous adipose tissue from Berkshires and F1 offspring. (E) Overall cluster heat map of the samples. Eighteen samples of subcutaneous adipose from six Ningxiang pigs, six Berkshires and six F1 pigs, respectively, were used to detect lipidomic profile.

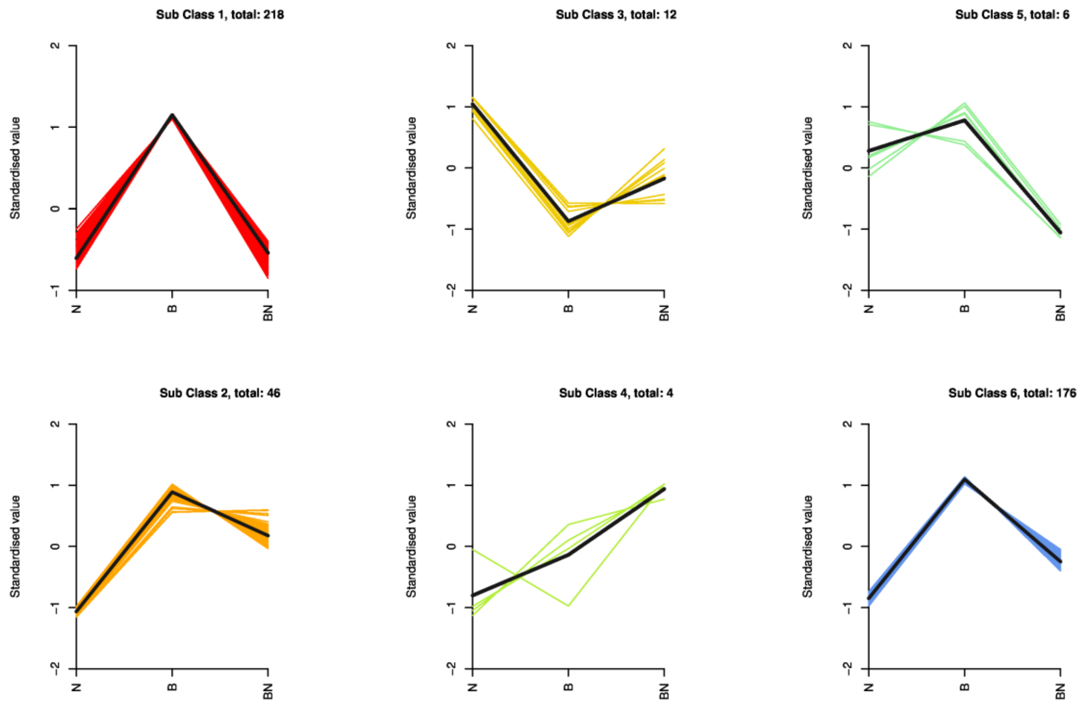

Figure S3. K-Means of SCLs. The horizontal ordinate represents sample groups, the Y-axis represents standardized relative lipid content, and Sub Class represents lipid class numbers with the same trend of change. Twelve samples of subcutaneous adipose from four Ningxiang pigs, four Berkshires and four F_1 pigs, respectively, were used to detect transcriptomic profile. B, Berkshires; N, Ningxiang pigs; BN, F_1 offspring.

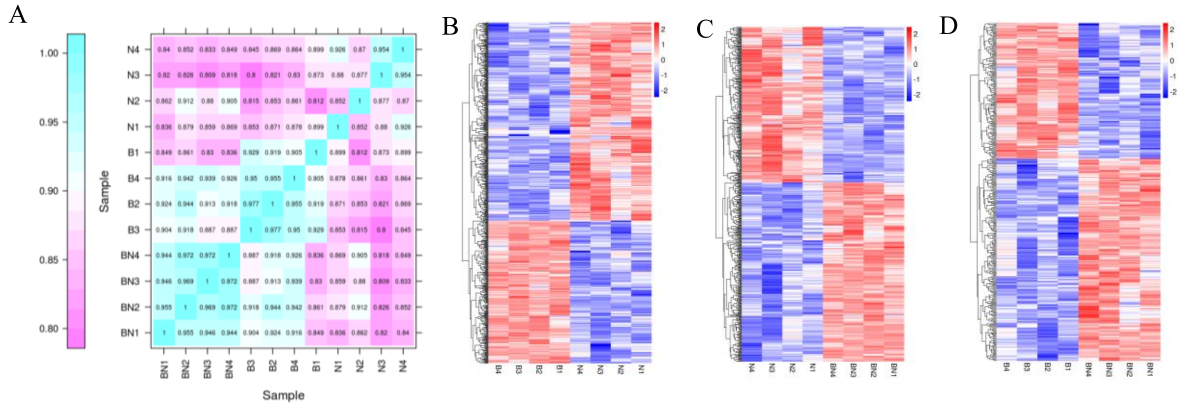

Figure S4. Transcriptome profiles diverse in the subcutaneous adipose tissue. (A) Transcriptome correlation analysis, B, Berkshire; N, Ningxiang pig; BN, F_1 offspring; (B-D) Cluster heat map of DEGs for the subcutaneous adipose tissue in the groups of Berkshires vs Ningxiang pigs (B), Ningxiang pigs vs F_1 offspring (C), Berkshires vs F_1 pigs (D). Twelve samples of subcutaneous adipose from four Ningxiang pigs, four Berkshires and four F_1 pigs, respectively, were used to detect transcriptomic profile. B, Berkshire; N, Ningxiang pig; BN. F_1 offspring.

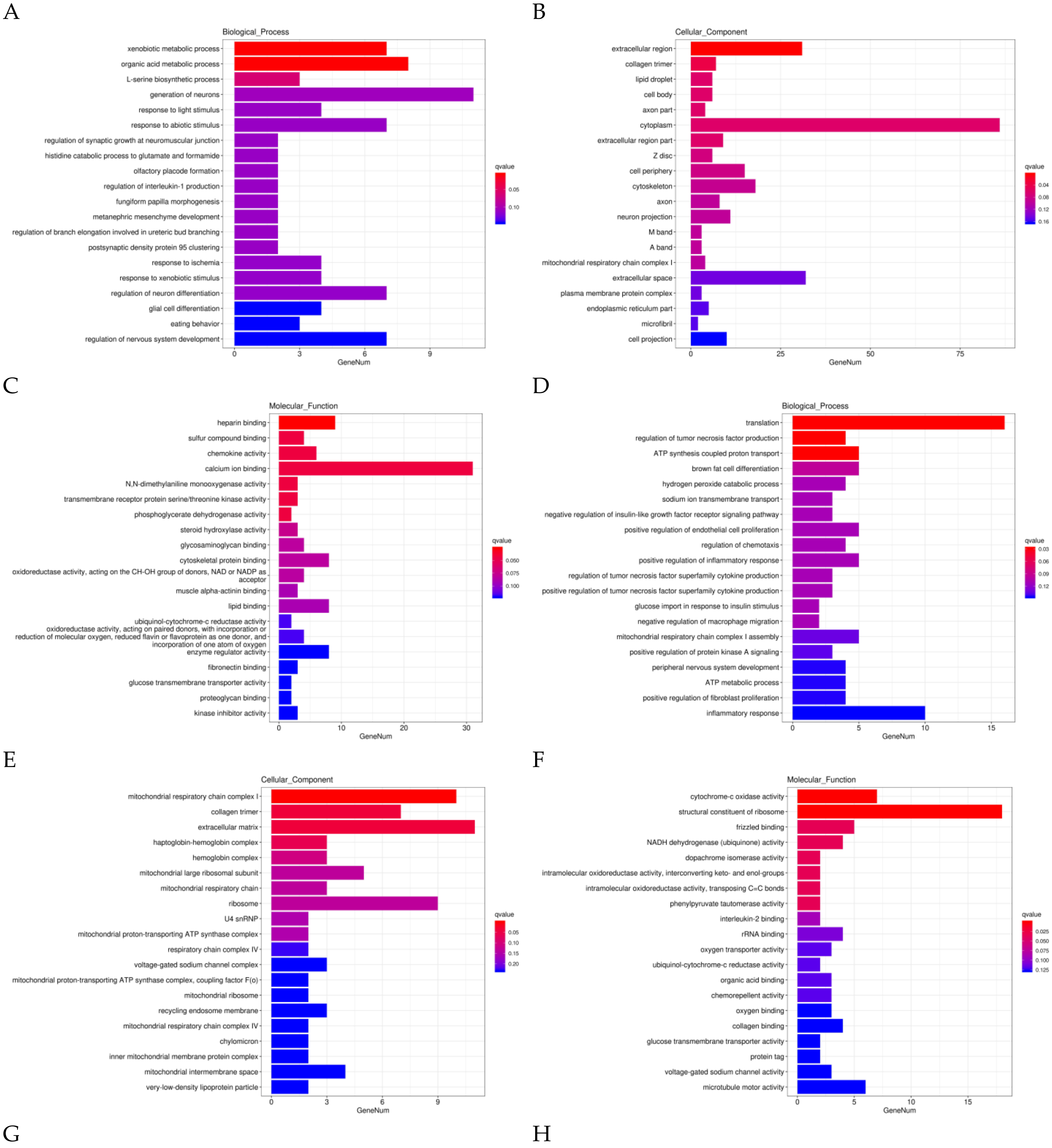

A
B
C
D
E
F
G
H
Biological_Process
xenobiotic metabolic process
organic acid metabolic process
L-serine biosynthetic process
generation of neurons
response to light stimulus
response to abiotic stimulus
regulation of synaptic growth at neuromuscular junction
histidine catabolic process to glutamate and formamide
olfactory placode formation
regulation of interleukin-1 production
fungiform papilla morphogenesis
metanephric mesenchyme development
regulation of branch elongation involved in ureteric bud branching
postsynaptic density protein 95 clustering
response to ischemia
response to xenobiotic stimulus
regulation of neuron differentiation
glial cell differentiation
eating behavior
regulation of nervous system development
GeneNum
qvalue
Cellular_Component
extracellular region
collagen trimer
lipid droplet
cell body
axon part
cytoplasm
extracellular region part
Z disc
cell periphery
cytoskeleton
axon
neuron projection
M band
A band
mitochondrial respiratory chain complex I
extracellular space
plasma membrane protein complex
endoplasmic reticulum part
microfibril
cell projection
GeneNum
qvalue
Molecular_Function
heparin binding
sulfur compound binding
chemokine activity
calcium ion binding
N,N-dimethylaniline monooxygenase activity
transmembrane receptor protein serine/threonine kinase activity
phosphoglycerate dehydrogenase activity
steroid hydroxylase activity
glycosaminoglycan binding
cytoskeletal protein binding
oxidoreductase activity, acting on the CH-OH group of donors, NAD or NADP as acceptor
muscle alpha-actinin binding
lipid binding
ubiquinol-cytochrome-c reductase activity
oxidoreductase activity, acting on paired donors, with incorporation or reduction of molecular oxygen, reduced flavin or flavoprotein as one donor, and incorporation of one atom of oxygen
enzyme regulator activity
fibronectin binding
glucose transmembrane transporter activity
proteoglycan binding
kinase inhibitor activity
GeneNum
qvalue
Biological_Process
translation
regulation of tumor necrosis factor production
ATP synthesis coupled proton transport
brown fat cell differentiation
hydrogen peroxide catabolic process
sodium ion transmembrane transport
negative regulation of insulin-like growth factor receptor signaling pathway
positive regulation of endothelial cell proliferation
regulation of chemotaxis
positive regulation of inflammatory response
regulation of tumor necrosis factor superfamily cytokine production
positive regulation of tumor necrosis factor superfamily cytokine production
glucose import in response to insulin stimulus
negative regulation of macrophage migration
mitochondrial respiratory chain complex I assembly
positive regulation of protein kinase A signaling
peripheral nervous system development
ATP metabolic process
positive regulation of fibroblast proliferation
inflammatory response
GeneNum
qvalue
Cellular_Component
mitochondrial respiratory chain complex I
collagen trimer
extracellular matrix
haptoglobin-hemoglobin complex
hemoglobin complex
mitochondrial large ribosomal subunit
mitochondrial respiratory chain
ribosome
U4 snRNP
mitochondrial proton-transporting ATP synthase complex
respiratory chain complex IV
voltage-gated sodium channel complex
mitochondrial proton-transporting ATP synthase complex, coupling factor F(o)
mitochondrial ribosome
recycling endosome membrane
mitochondrial respiratory chain complex IV
chylomicron
inner mitochondrial membrane protein complex
mitochondrial intermembrane space
very-low-density lipoprotein particle
GeneNum
qvalue
Molecular_Function
cytochrome-c oxidase activity
structural constituent of ribosome
frizzled binding
NADH dehydrogenase (ubiquinone) activity
dopachrome isomerase activity
intramolecular oxidoreductase activity, interconverting keto- and enol-groups
intramolecular oxidoreductase activity, transposing C=C bonds
phenylpyruvate tautomerase activity
interleukin-2 binding
rRNA binding
oxygen transporter activity
ubiquinol-cytochrome-c reductase activity
organic acid binding
chemorepellent activity
oxygen binding
collagen binding
glucose transmembrane transporter activity
protein tag
voltage-gated sodium channel activity
microtubule motor activity
GeneNum
qvalue

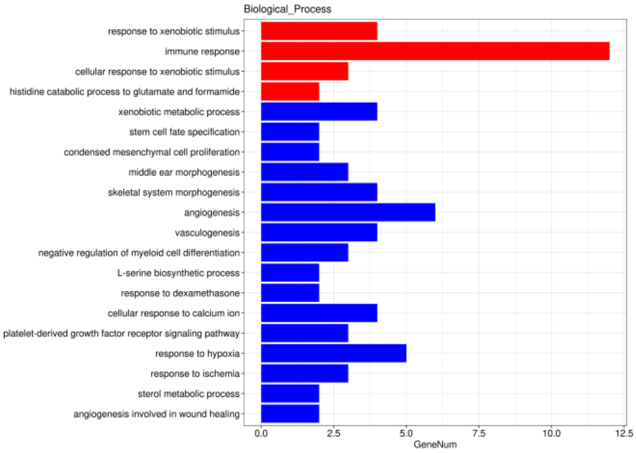

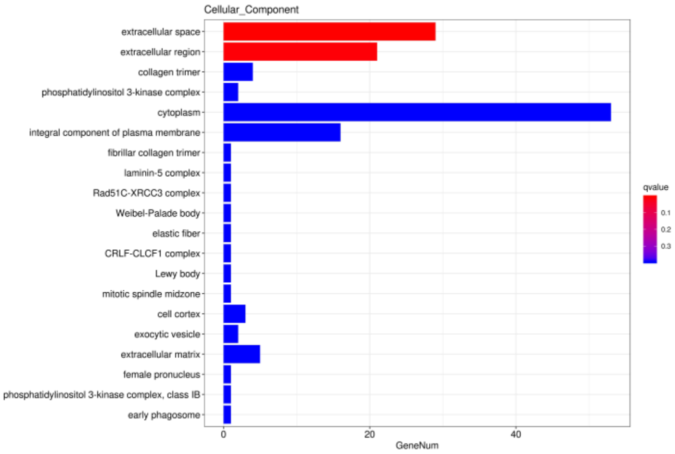

I

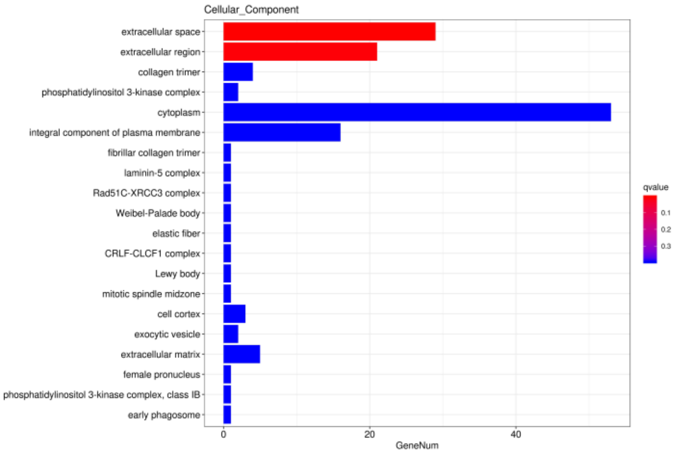

Figure S5. A partial list of bioinformatics analysis results using DEGs of the subcutaneous adipose tissue. (A-C) The column diagrams for the analysis of biological processes (A), cellular components (B), and molecular functions (C) of DEGs in Berkshires vs. Ningxiang pigs; (D-F) The column diagrams for the analysis of biological processes (D), cellular components (E), and molecular functions (F) of DEGs in Ningxiang pigs vs. F_1 offspring; (G-I) The column diagrams for the analysis of biological processes (G), cellular component (H), and molecular function (I) of DEGs in Berkshires vs. F_1 offspring. Twelve samples of subcutaneous adipose from four Ningxiang pigs, four Berkshires and four F_1 pigs, respectively, were used to detect transcriptomic profile.

A

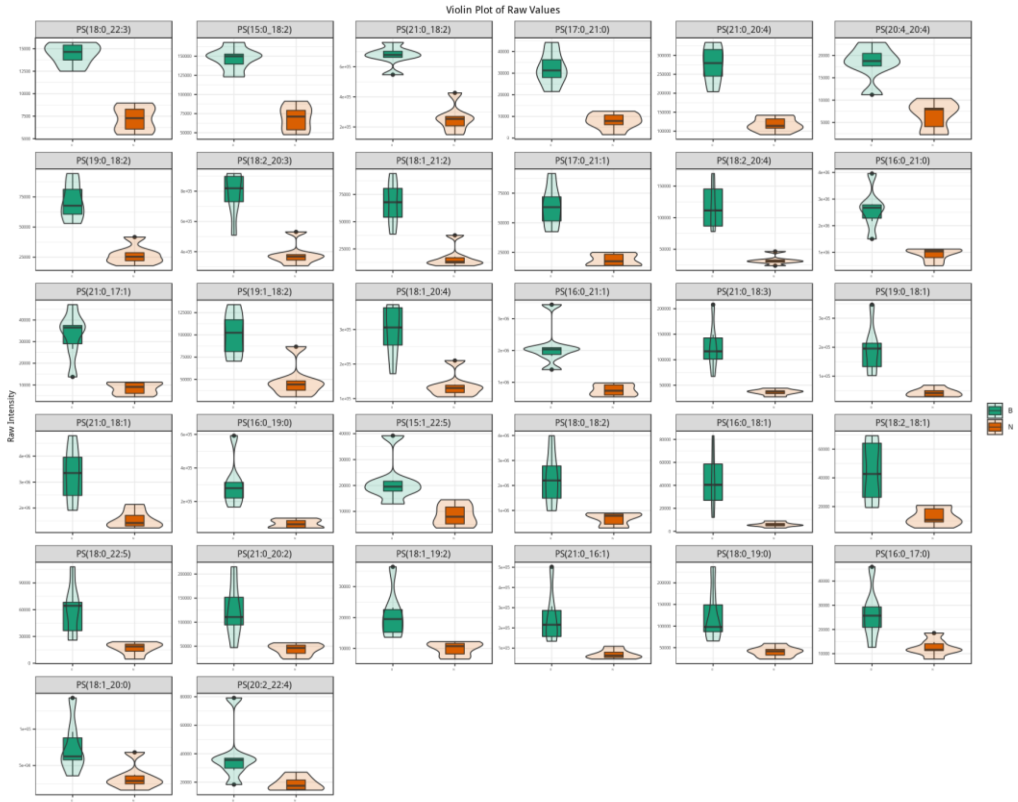

B

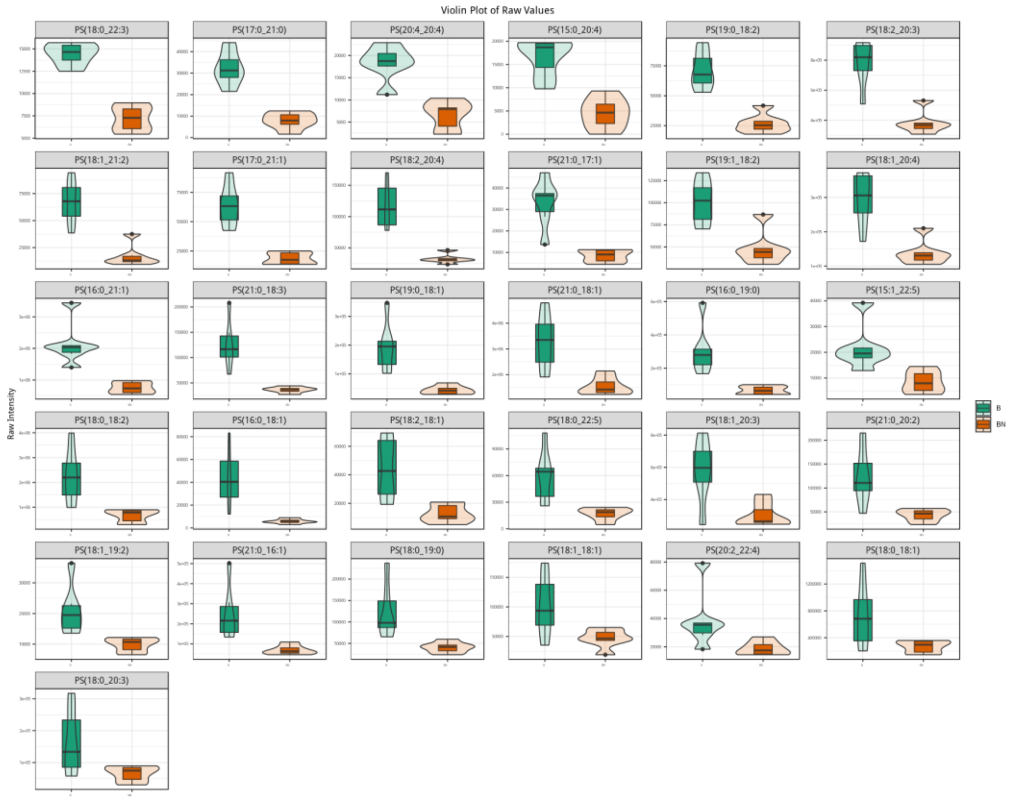

Figure S6. The violin chart of the SCLs related to kO00260 pathway. The violin chart of Phosphatidylserines in Berkshires vs. Ningxiang pigs (A) and Berkshires vs F_1 offspring (B), respectively. B, Berkshires; N, Ningxiang pig; BN. F_1 offspring. .

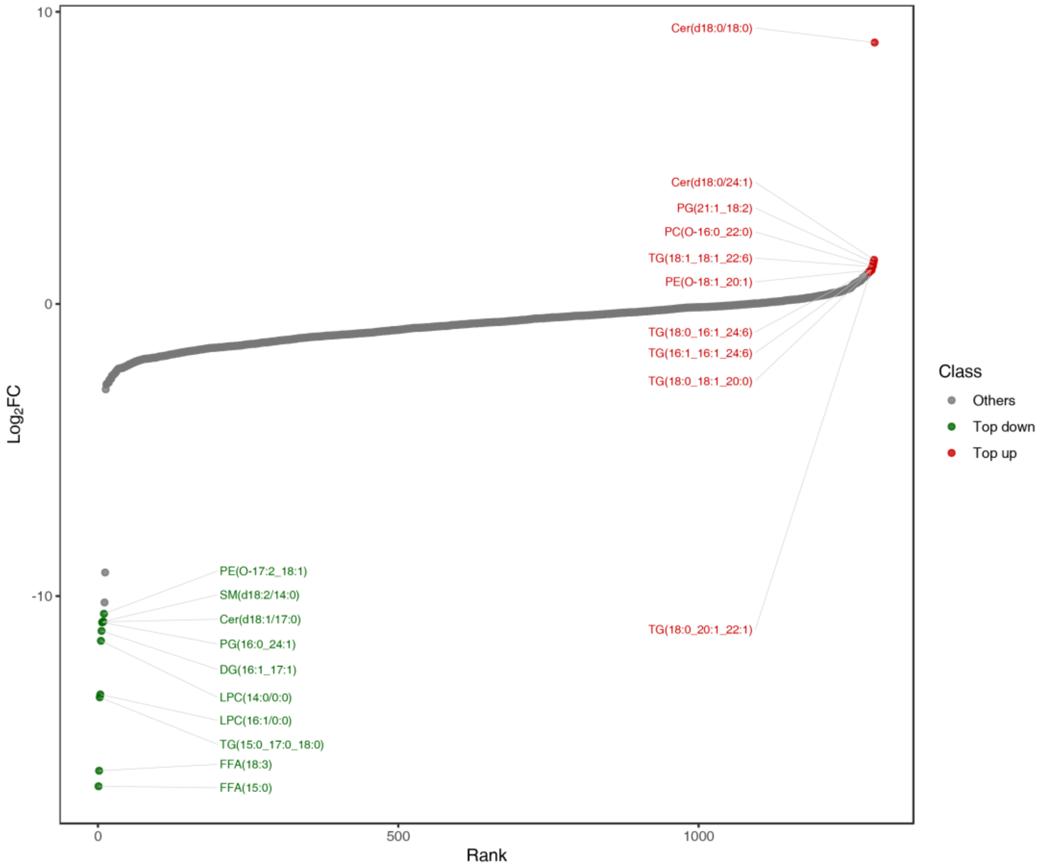

Figure S7. Dynamic distribution map of lipid content differences in the subcutaneous adipose tissue from Berkshires vs. Ningxiang pigs. The horizontal ordinate represents the different multiples of substances, the Y-axis represents the log_2FC. Each point represents a substance, green dots represent the substances in the top 10 of the downward ranking, and red dots represent the substances in the top 10 of the upward ranking.

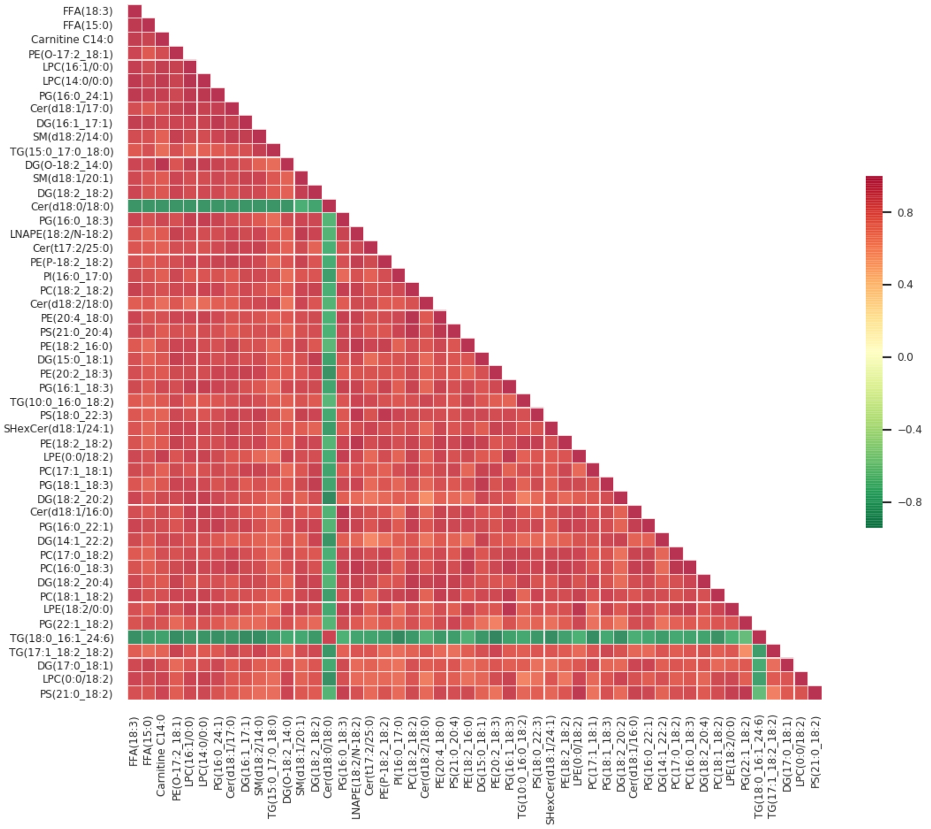

Figure S8. The correlation heat map for significantly different lipids with the top VIP value in the subcutaneous adipose tissue in Berkshires vs. Ningxiang pigs. Red represents positive correlations, green represents negative correlations.

A

B

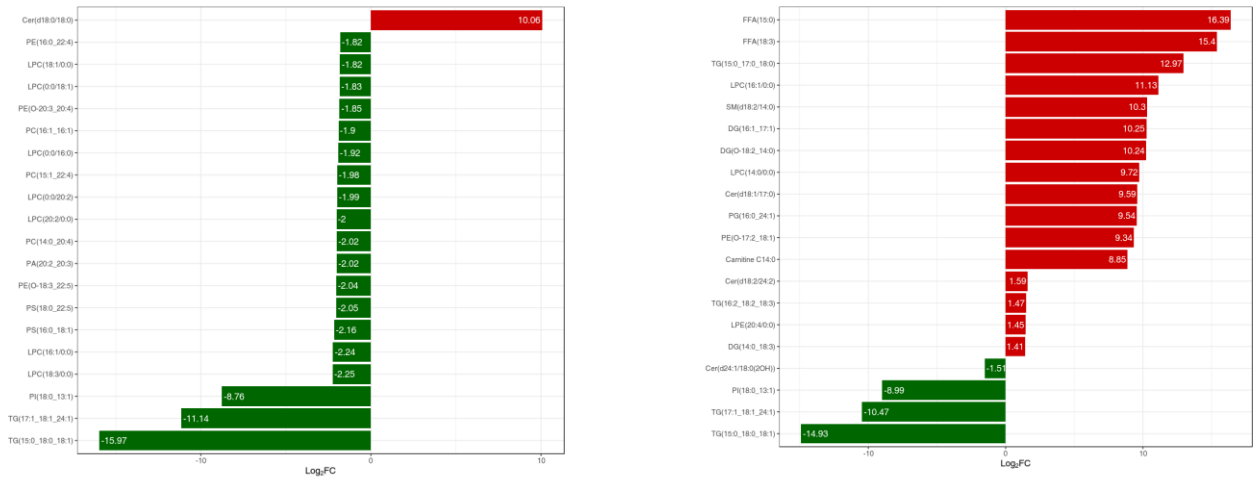

Figure S9. Bar chart of different lipids in the subcutaneous adipose tissue in Berkshires vs. F_1 offspring (A) and in Ningxiang pigs vs. F_1 offspring (B). The horizontal ordinate represents the log_2FC of different lipids. The Y-axis represents differential lipids. Red represents an increase in lipid content, while green represents a decrease in lipid content.

Table S1. DEGs in Berkshires vs. Ningxiang pigs enriched in partial KEGG pathways ($P < 0.05$).

Gene name	FPKM		FDR	Log_2FC	regulate	Gene annotated
	BKX	NX				
**kO00260 glycine, serine and threonine metabolism**						
PHGDH	28.12	4.17	$2.27E^{-11}$	-2.64	down	phosphoglycerate dehydrogenase
LOC100156167	8.84	1.44	$3.84E^{-7}$	-2.41	down	phosphoglycerate dehydrogenase like protein
SHMT1	86.01	26.98	$9.92E^{-4}$	-1.56	down	glycine hydroxymethyltransferase
PSPH	12.56	52.64	$9.42E^{-6}$	2.21	up	phosphoserine phosphatase
LOC110256000	3.37	19.40	$9.12E^{-08}$	2.49	up	membrane primary amine oxidase-like
**KO00590 arachidonic acid metabolism**						
CYP2B22	69.83	17.85	$1.32E^{-3}$	-1.85	down	cytochrome P450 family 2 subfamily B22
LOC110255237	6.63	22.28	$2.09E^{-4}$	1.86	up	cytochrome P450 4F6-like
GGT5	8.43	23.69	$1.70E^{-3}$	1.54	up	gamma-glutamyltranspeptidase
PTGES	3.91	13.12	$4.47E^{-8}$	1.86	up	microsomal prostaglandin-E synthase 1
SAL1	25.78	134.40	$3.37E^{-6}$	2.45	up	salivary lipocalin
GPX1	334.05	1411.35	$3.60E^{-7}$	2.20	up	glutathione peroxidase 1
GPX3	2304.61	1055.47	$4.01E^{-7}$	-1.01	down	
**KO00061 fatty acid biosynthesis**						
FASN	1320.46	2689.93	$1.19E^{-3}$	1.15	up	fatty acid synthase
ACACA	26.60	73.44	$5.13E^{-9}$	1.58	up	acetyl-CoA carboxylase
CBR4	3.87	7.46	$9.83E^{-6}$	1.06	up	carboxyl reductase 4
MCH	0.19	0.82	$6.33E^{-3}$	2.20	up	medium chain acyl hydrolase
**KO01212 fatty acid metabolism**						
EHHADH	8.54	16.07	$2.70E^{-3}$	1.03	up	enoyl-CoA hydratase
FASN	1320.46	2689.93	$1.19E^{-3}$	1.15	up	fatty acid synthase

Gene name	FPKM		FDR	Log_2FC	regulate	Gene annotated
	BKX	NX				
ACACA	26.60	73.44	$5.13E^{-9}$	1.58	up	acetyl-CoA carboxylase
CBR4	3.87	7.46	$9.83E^{-6}$	1.06	up	carboxyl reductase 4
HACD	98.60	42.53	$7.91E^{-6}$	-1.10	down	very long-chain (3R)-3-hydroxyacyl-CoA dehydratase
ELOV6	182.38	428.54	$9.82E^{-5}$	1.36	up	very long chain fatty acids protein 6
SCD	1184.93	3625.88	$5.31E^{-5}$	1.75	up	stearoyl-CoA desaturase
**kO00561 glycerolipid metabolism**						
GK	23.72	8.19	$5.06E^{-9}$	-1.37	down	glycerol kinase
MGLL	192.95	383.22	$7.45E^{-10}$	1.04	up	monoglyceride lipase
LPIN1	127.44	44.92	$5.42E^{-4}$	-1.40	down	lipin 1
DGKB	3.62	1.58	$1.28E^{-3}$	-1.07	down	diacylglycerol kinase beta
DGAT2	109.71	518.44	$8.38E^{-20}$	2.36	up	diacylglycerol O-acyltransferase 2
**ko04070 phosphatidylinositol signaling system**						
NTMR7	0.26	1.43	$1.36E^{-3}$	2.54	up	myotubularin-related protein 7
PLCB1	4.49	2.06	$1.14E^{-6}$	-1.01	down	phospholipase C beta 1
PIK3C2G	9.04	3.31	$4.24E^{-14}$	-1.30	down	phosphatidylinositol-4-phosphate 3-kinase catalytic subunit type 2 gamma
DGKB	3.62	1.58	$1.28E^{-3}$	-1.07	down	diacylglycerol kinase beta
PLCE1	0.45	0.19	$4.25E^{-3}$	-1.11	down	phospholipase C epsilon 1
CALML5	2.09	0.13	$6.52E^{-6}$	-3.91	down	calmodulin like 5
LOC102157965	0.15	1.53	$2.35E^{-4}$	4.36	up	parvalbumin beta-like
**kO04973 carbohydrate digestion and absorption**						
ATP1A1	83.22	34.41	$2.93E^{-7}$	-1.17	down	sodium/potassium-transporting ATPase subunit alpha1
HK3	4,41	9.39	$6.21E^{-3}$	1.24	up	hexokinase3
SLC2A5	12.86	32.20	$5.91E^{-3}$	1.44	up	Solute carrier family 2 member 5
PLCB1	4.49	2.06	$1.14E^{-6}$	-1.01	down	phospholipase C beta 1
**kO04714 thermogenesis**						
MGLL	192.95	383.22	$7.45E^{-10}$	1.04	up	acylglycerol lipase
ATP5MC1	79.10	148.15	$7.42E^{-3}$	1.02	up	ATP synthase membrane subunit c locus 1
UQCR10	63.91	150.00	$2.65E^{-4}$	1.35	up	ubiquinol-cytochrome c complex III subunit X
NDUFA3	11.52	25.38	$8.14E^{-3}$	1.26	up	NADH:ubiquinone oxidoreductase subunit A3
UQCRQ	47.93	108.01	$2.28E^{-6}$	1.29	up	ubiquinol-cytochrome c reductase complex III subunit VII
COX7C	134.91	329.31	$2.04E^{-3}$	1.34	up	cytochrome c oxidase subunit 7C
NDUFA11	45.34	85.17	$9.02E^{-5}$	1.03	up	NADH:ubiquinone oxidoreductase subunit A11
NDUFB2	39.84	81.82	$2.27E^{-3}$	1.20	up	NADH:ubiquinone oxidoreductase subunit B2
NDUFA2	33.06	62.73	$7.84E^{-8}$	1.04	up	NADH:ubiquinone oxidoreductase subunit A2
COX17	41.24	105.00	$4.63E^{-7}$	1.47	up	cytochrome c oxidase copper chaperone COX17
UQCR11	1.27	7.07	$2.71E^{-3}$	2.58	up	ubiquinol-cytochrome c reductase, complex III subunit XI

Table S2. Primers for real-time fluorescent quantitative PCR.

Gene	Primer sequence (5'-3')
β-actin	sense: CCAGGTCATCACCATCGG<br>Antisense: CCGTGTTGGCGTAGAGGT
FASN	sense: GGGCCCAGCATCACCATAGACA<br>Antisense: GTTCGTGCCCGCATTGAGGAT
SCD	sense: CTTCCTGATCATTGCCAACA<br>Antisense: GCAAACCACCCTTCTCTTTG
PSPH	sense: GATGCTGTGTGTTTTGATGTTGAC<br>Antisense: CTTGACTTGTTGCCTGATCACATT
ACACA	sense: GTCCTCTGCCAGTTTCCC<br>Antisense: TCCATCACCACAGCCTTC
ELOV6	sense: CCGGAAGTTTGCCATGTTCA<br>Antisense: GCAGAAGAGCACAAGGTAGC
HACD2	sense: CAGACCGGAGCTCTTCTGG<br>Antisense: TGTCTTCATTCTGGACCTCTCG
GPX1	sense: CAAGAATGGGGAGATCCTGA<br>Antisense: GATAAACTTGGGGTCGGTCA
GPX3	sense: CTCGGAGATTCTGTCCACTCTCA<br>Antisense: CCGTTCACGTCCCCTTTCT
LPIN1	sense: GATCATCTCCGACATTGACG<br>Antisense: TGGTACAGCTTGGCTATGC
MGLL	sense: TATGAGGGTGCCTACCAC<br>Antisense: GTCCTTTGGGAGACCCAT
CYP2B22	sense: AGTGTGGAGAAGCACCGTGAA<br>Antisense: GCAAAGAAGAGCGAAAGGACAGT
DGAT2	sense: TGGCTCTTTCCATTTCATCC<br>Antisense: CGTAAGCACGATCAGAACGA
